# Discrimination of large maltooligosaccharides from isobaric dextran and pullulan using ion mobility mass spectrometry

**DOI:** 10.1002/rcm.6771

**Published:** 2014-12-05

**Authors:** Abdul M Rashid, Gerhard Saalbach, Stephen Bornemann

**Affiliations:** Department of Biological Chemistry, John Innes CentreNorwich Research Park, Norwich, NR4 7UH, UK

## Abstract

**RATIONALE:**

Ion mobility mass spectrometry (IMMS) has previously been shown to resolve small isobaric oligosaccharides, but larger alpha-oligoglucans are also abundant in biology and are of industrial importance. If conformational differences between such isomers are retained in the gas phase, IMMS could be used to address questions in biology and industry.

**METHODS:**

Negative mode electrospray ionization (ESI) travelling-wave IMMS was used to resolve large isobaric α-glucan ions on the basis of their different gas-phase conformations. α,ω-Dicarboxy-terminated polystyrene was used to calibrate the instrument allowing the collision cross-sections (CCSs) of ions to be determined.

**RESULTS:**

α-1,4-Linked maltooligosaccharides with a degree of polymerisation of up to 35 could be discriminated from α-1,6-linked dextran and α-1,4/1,6-linked pullulan using IMMS. Fragmentation spectra of ions separated by IMMS could also distinguish isomers. Two conformational isomers of maltohexaose were resolvable by IMMS, likely reflecting extended and V6 helical conformations. IMMS was also able to identify a product within a mixture of maltooligosaccharides treated with the potential anti-tuberculosis drug target *Mycobacterium tuberculosis* GlgB branching enzyme.

**CONCLUSIONS:**

Biological samples of complex isobaric oligosaccharides can be analysed using IMMS in the negative mode providing facile analyses and high sensitivity without the need for either derivatisation or chromatographic separation. © 2013 The Authors. *Rapid Communications in Mass Spectrometry* published by John Wiley & Sons Ltd.

α-Glucans are not only abundant in biological systems, but are also of industrial importance. For example, starch, with its α-1,4-linked amylose component, is a carbon storage compound, major dietary component and industrial feedstock with uses in the biofuel, pharmaceutical, textile and paper industries on a 10^9^ tons year^–1^ scale.^1^,^2^ α-Glucans related to starch also exist in bacteria and are involved in carbon storage and immune evasion.^3^ α-1,6-Linked dextran and α-1,4- and α-1,6-linked pullulan are associated with microbial biofilms and have uses in foods, medicine, cosmetics and research.^4^,^5^ The composition, biosynthesis, modification and degradation of such molecules are not fully understood and this is largely because the analysis of complex biological α-glucan samples with a range of degrees of polymerisation (DP; i.e. the number of glucose units) can be challenging. Chromatography with or without derivatisation followed by compound identification is a well-established strategy that can provide useful information, but at the expense of time and sensitivity. Despite the versatility of traditional mass spectrometric methods, isobaric carbohydrates such as α-glucans add additional challenges.

Several new approaches have been developed to tackle isobaric carbohydrates such as ion mobility mass spectrometry (IMMS) and energy-resolved collision-induced dissociation.^6^ IMMS provides a two-dimensional (2D) method of separating ions on the basis of shape through mobility within a gas-filled drift tube and then *m/z*. Collision cross-sections (CCSs) of ions can be determined directly with some instruments and estimated with others through calibration with standards. Most reported analyses are in the positive mode with alkali metal coordinated ions,^7^–^16^ with relatively few in the negative mode.^17^ Recent examples of IMMS being able to differentiate isobaric oligosaccharides include *N*-linked glycans associated with proteins,^8^–^12^ glycosaminoglycans^18^ and other small oligosaccharides^14^ including a few glucans with a DP of up to 5.^13^,^15^–^17^ It has been possible to resolve isobaric carbohydrates with different primary structures, branching patterns and anomeric/positional glycosidic linkages. More recent advances included combining IMMS with tandem mass spectrometry^14^,^17^,^19^ and the development of tandem IMMS.^13^ However, there are no reports of a systematic analysis of larger linear homo- and hetero-oligomeric α-glucans using IMMS establishing whether conformational differences in aqueous solution are retained with such large molecules in the gas phase.

We now show that linear homo- and hetero-oligomeric α-glucans with a DP of up to 35, which differ in only their content of α-1,4- and α-1,6-linkages, can be resolved using IMMS in the negative mode with ion mobilities consistent with known molecular conformations in solution. In addition, calibration with α,ω-dicarboxy-terminated polystyrene^20^ allows the CCS of each species to be estimated. Furthermore, the ability to identify with IMMS a product of the *Mycobacterium tuberculosis* GlgB branching enzyme within a complex maltooligosaccharide mixture is also demonstrated.

## EXPERIMENTAL

### Materials

α,ω-Dicarboxy-terminated polystyrene (number average molecular mass [M_n_] 800, weight average molecular weight [M_w_] 1120 and polydispersity index [PDI] M_w_/M_n_ 1.4) was supplied by Polymer Source and decadeoxythymidine was purchased from Invitrogen. Individual maltooligosaccharides (DP 2–7, **2–7**), dextran 5000, pullulan 1300 and bovine pancreas oxidised insulin chain A were purchased from Sigma Aldrich Chemical Co. D-Panose **3n** and maltooctaose **8** were purchased from Carbosynth Ltd, 6^3^-α-D-glucosyl-maltotriose **4n** and 6^3^-α-D-glucosyl-maltotriosyl-maltriose **7n** were purchased from Megazyme Ltd, and amylose 2800 (average molecular weight; containing a range of maltooligosaccharides of DP 2 to 35) was purchased from TCI Europe N.V.

### Ion mobility mass spectrometry (IMMS)

Solutions were prepared of polystyrene (50 μM) in methanol/chloroform (70:30) with a trace of ammonia, and of decadeoxythymidine, insulin (1 mM) and glucans (1 μM) in water and diluted 100-fold with water/methanol (1:1). Samples were injected into a Synapt G2 HDMS mass spectrometer (Waters Corp., Manchester, UK) via a capillary sprayer at a flow rate of 5 μL min^–1^. Ions produced by ESI were introduced into the drift tube in travelling-wave mobility mode. The sampling cone and capillary voltages were held at 35 and 3.6 kV, respectively. The IMMS nitrogen and helium cell gas flows were set to 90 and 180 mL min^–1^, respectively. The IMMS travelling-wave velocity and height was set to 600 m/s and 40 V, respectively. Spectra were recorded in the negative resolution mode. The variation in mode drift times for a given sample in replicate experiments was ≤1% and the maximum width at half height of any given signal was typically 1–3% of the absolute mode drift time. Shifts in drift times for a given species of 1–2% were observed when comparing samples of single compounds with complex mixtures.

### Reduction of glucan and hydron exchange

Glucan was reduced according to a published procedure,^21^ with modifications. A solution of sodium borohydride (1–2 mg) in water (10 μL) was added to a solution of the glucan (10 mg) in water (50 μL). The reaction mixture was incubated at 21 °C for 1 h, acidified with dilute acetic acid, and treated with absolute ethanol (5 volumes). The resulting precipitated material was isolated by centrifugation and dried. Hydrons were exchanged in maltohexaose (1 mg) by dissolution in deuteriated water (25 μL) and incubation at 21 °C for 24 h. A 1 μL sample of the product was diluted 50-fold with 1:1 CD_3_OD/D_2_O for IMMS.

### Collision cross-section (CCS)

CCSs were obtained from drift time measurements with α,ω-dicarboxy-terminated polystyrene as a negative ion calibrant.^20^ The calibrant has two carboxyl end groups allowing both singly and doubly charged ion data to be obtained. Calibration curves for singly or doubly charged species were obtained using linear least-squares fitting. The standard error associated with estimating the CCS from the linear calibration curve was <1% for both interpolated and extrapolated values.^22^ The CCS of species with z ≥3 was estimated using Waters Driftscope software, which calculates appropriate calibration curves from the measured calibration curves.

### Amylose 2800 branching by GlgB

The expression in *Escherichia coli* and purification of recombinant His-tagged *M. tuberculosis* H37Rv GlgB was carried out using a strategy used with other glucan-processing enzymes.^23^ Details will be provided elsewhere. A reaction mixture (100 μL) containing 20 mM sodium phosphate buffer, pH 7.4, 20 mM NaCl, 2 mg amylose 2800 and 1 µg purified GlgB was incubated at 30 °C for 1 h. The reaction was terminated by heating at 95 °C for 5 min. The reaction mixture was treated with absolute ethanol (5 volumes) and the resulting precipitated material was isolated by centrifugation and dried under vacuum.

## RESULTS AND DISCUSSION

### IMMS of maltooligosaccharides

Many MS studies of oligosaccharides have been carried out in the positive mode with sodiated ions with or without derivatisation such as with boronic acid.^24^ However, MS of the α-glucans used in the present study gave stronger signals with [M–H]^–^ ions in the negative mode. MS of individual or mixed α-1,4-linked maltooligosaccharides gave ions with DP 2–10 (compounds **2**, **3**, **4**, **5**, **6**, **7**, **8**, **9** and **10** in Table 1) in their singly charged monomeric form. Compounds **2** and **3**, and to some extent compound **4**, were prone to form singly charged dimers while compounds **5** and **6** did not form dimers under these conditions. Interestingly, compounds **7**, **8** and to some extent **4** formed doubly charged dimers. Dimerisation does not appear to be an exclusively gas-phase phenomenon because it has also been observed in aqueous solution.^25^

**Table 1:** Negative ion mobility and collision cross-section (Ω) of glucans

	Glucan	DP	Ion	*m/z*	z	Drift time (ms)	Ω (Å^2^)
**2**	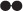	2	Monomer	341.1	1	3.02	116
			Dimer	683.2	1	5.98	174
**3**	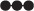	3	Monomer	503.2	1	4.53	146
			Dimer	1007.4	1	7.36	200
**3n**	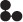	3	Monomer	503.2	1	4.18	139
			Dimer	1007.4	1	7.78	209
**4**	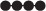	4	Monomer	665.2	1	5.30	160
			Dimer	1331.4	1	10.12	254
			Dimer	665.2	2	6.30	417
**4n**	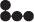	4	Monomer	665.2	1	5.57	166
			Dimer	1331.4	1	10.67	265
			Dimer	665.2	2	6.38	422
**5**	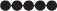	5	Monomer	827.3	1	6.81	190
**6a**	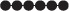 isomer a	6	Monomer	989.4	1	7.78	209
**6b**	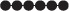 isomer b	6	Monomer	989.4	1	8.26	218
**6r**	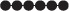 reduced	6	Monomer	991.4	1	8.23	217
**6d**	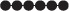 deuteriated	6	Monomer	1007.5	1	7.94	212
**7**	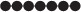	7	Monomer	1151.4	1	9.60	244
			Dimer	1151.4	2	5.42	366
**7n**	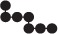	7	Monomer	1151.4	1	8.88	230
**8**	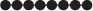	8	Monomer	1313.5	1	10.74	266
			Dimer	1313.5	2	6.17	409
**9**	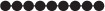	9	Monomer	1475.5	1	11.57	282
**10**	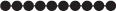	10	Monomer	1637.5	1	12.61	302
	GlgB substrate isomer	11	Monomer	899.3	2	4.38	305
	GlgB product isomer	11	Monomer	899.3	2	4.94	338

Filled circles represent glucose rings. Horizontal and vertical bonds represent α-1,4- and α-1,6-glycosidic linkages, respectively, where the reducing end is at the right-hand end.

Compounds **9** and **10** were constituents of amylose 2800 while all other numbered compounds were authentic pure compounds.

The GlgB product isomer was obtained by treating amylose 2800 with *M. tuberculosis* GlgB branching enzyme.

It would be expected that mobility would decrease as the DP increased. This was indeed observed because the drift times increased as the DP increased. Similarly, the drift times of singly charged dimers were larger than those of the corresponding singly charged monomers. Thus, it was possible to determine the mobility of oligo-glucans in the negative mode.

### IMMS separation of two conformational isomers of maltohexaose

With one exception, each maltooligosaccharide gave one monomeric singly charged species in the IMMS conditions used. This contrasts with other studies with reducing sugars, which were complicated by the presence of α and β anomers and/or pyranose, furanose and ring-opened conformations.^13^–^15^ Such phenomena can be negated by derivatisation to either alditols or methyl glycosides, but this was unnecessary in the present study.

**Figure 1:**
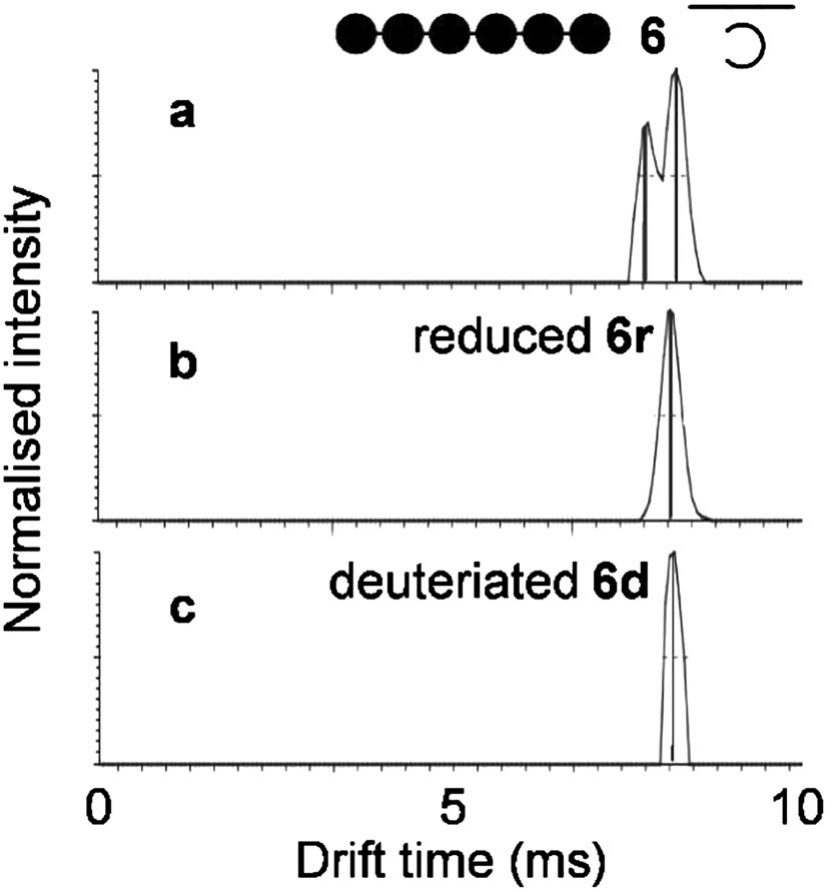
IMMS of maltooligosaccharide with DP 6 (6a and 6b, *m/z* 989) showing two conformational isomers with different ion mobility (a). The straight and curved lines are cartoons of likely extended and helical conformations. Only one isomer was detected after either reduction (b, 6r, *m/z* 991) or deuteriation (c, 6d, *m/z* 1007).

The conspicuous exception was maltohexaose **6**, which was resolvable into two isomers (Table 1 and Fig. 1). When **6** was reduced with sodium borohydride, the product **6r** had an *m/z* heavier by 2, consistent with the reduction of the two anomers to maltohexitol, and was no longer resolvable into two isomers. Compound **6** was also treated with deuterium oxide to replace exchangeable hydrogens with deuterium and potentially strengthen any hydrogen-bonding interactions and/or perturb the equilibria between anomeric and ring forms. The resulting deuteriated compound **6d** had 18 hydrons exchanged, which presumably comprised 18 of the 20 hydrons associated with hydroxyl groups. This compound **6d** was no longer resolvable into two isomers (Fig. 1).

At face value, it would seem as though the two isomers of maltohexaose could reflect different anomeric or ring forms. However, this seems unlikely because all of the other maltooligosaccharides gave only one species each and the smaller compounds might be expected to be disproportionately affected. Similarly, the possibility that alternative locations of charge could give different conformations of an ion^14^ seems unlikely to be confined to maltohexaose.

Maltooligosaccharides are known to form rigid left-handed V6 helices with six sugar units per turn in aqueous solution.^26^ This involves an O(6)–O(2) intra-helical hydrogen-bonding interaction between every sixth sugar residue. A transition from an extended to a helical conformation occurs when the DP reaches 6, when the first turn can be completed.^25^ Interestingly, maltohexaose is unusual in that it can form two types of dimers in aqueous solution through either the sideways association of extended molecules or the stacked association of helical molecules. This implies that making the first hydrogen bond along the axis of the helix provides only marginal stabilisation of the helical conformation and that additional interactions associated with a higher DP push the equilibrium more strongly towards helix formation. It is therefore likely that the formation of the first intra-helical hydrogen bond could be affected by the conformation of the reducing end sugar ring and the introduction of deuterium. This suggests that both extended and helical conformations of **6** exist in the gas phase and that they are resolvable by IMS under the conditions used, provided the pyranose ring is intact and the hydroxyls contain hydrons.

### Determination of collisional cross-section (CCS)

The calibration of the CCS for glycans has been described in the positive mode but few examples exist in the negative mode. The best option that reasonably spans the range associated with singly and doubly charged oligosaccharide ions of CCS between ∼130 and ∼390 Å^2^ in the negative mode appears to be with α,ω-dicarboxy-terminated polystyrene.^20^ This approach was used (Supplementary Table S1, see Supporting Information) and, in order to validate the calibration and establish whether it was possible to extrapolate it to ∼640 Å^2^, the negative ion mobility of two other known compounds was determined; oxidised insulin chain A from bovine pancreas^27^ and decadeoxythymidine.^28^ The values of CCS were similar to those reported previously (Supplementary Tables S2 and S3, see Supporting Information), where intra-molecular charge repulsion and consequent conformational changes are thought to cause the increase in CCS with increasing values of *z*.^27^,^28^ In conclusion, the calibration using polystyrene appeared sufficiently robust to determine the CCSs of oligosaccharides with a wide range of DP.

With CCS being a function of ion mobility, the CCS of maltooligosaccharides increased with DP as expected. The footprint of a glucose ring has been reported to be ∼50 Å^2^^29^,^30^ so the CCS of maltose (compound **2** with DP 2, Table 1) of 106 Å^2^ was consistent with double this value as expected. However, the CCS did not continue to double either as the DP doubled or when singly charged dimers were formed, illustrating the non-linear relationship between CCS and the number of sugar rings. This is presumably due to the influence of the conformation of the linear oligosaccharides. As with the protein and nucleotide standards, the CCS of maltooligosaccharides increased as *z* increased, consistent with the conformation of the glucans becoming more extended due to charge repulsion. It is noteworthy that, although it was possible to calculate calibration curves for one charge state from curves of another using the Driftscope software, more reliable data was obtained from experimentally determined calibration data with the appropriate charge state, as reported by others.^10^

### IMMS separation of linear homo-oligomeric α-1,4- and α-1,6-linked glucans

**Figure 2:**
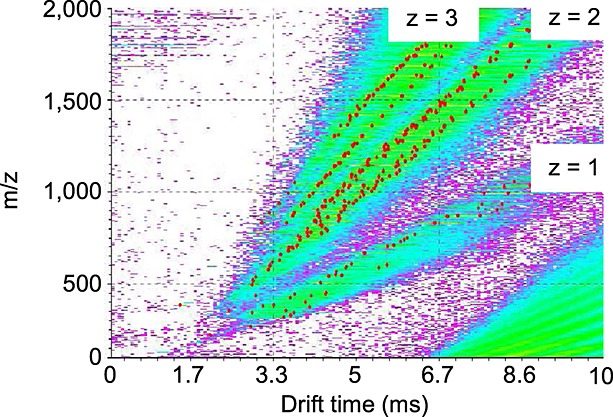
ESI T-Wave IMMS spectra of a mixture of dextran 5000 and amylose 2800 (maltooligosaccharides) showing singly, doubly and triply charged ion clouds. Red dots highlight significant ions. The separation of ions derived from amylose (lower drift time to the left) and dextran (higher drift time to the right) of the same *m/z* and charge was observed.

While α-1,4-linked maltooligosaccharides form rigid helical structures, with each disaccharide unit having the somewhat restricted conformation of maltose, α-1,6-linked dextrans are known to form more extended random coil structures, given the greater freedom provided by the primary 6 position of the sugar ring.^31^ It was therefore of interest whether this difference in conformation could be detected using IMMS with larger oligomers. Samples of amylose 2800 (maltooligosaccharides) and dextran 5000, which both contained a range of DP (2 to at least 35), were analysed either separately (Tables 1, 2, 3 and 4) or in a mixture (Fig. 2). Ion clouds with 1, 2 and 3 negative charges were observed with clear separation of the maltooligosaccharide and dextran ions with identical *m/z*. Ions with a low DP had a relatively low abundance, but were nevertheless more easily detected in the singly charged state (Table 2 and Fig. 2). Doubly charged ions were more readily detected and those with a moderate DP were particularly abundant (Table 3 and Fig. 2). Molecules with the highest DP carried predominantly three charges (Table 4 and Fig. 2). In general, maltooligosaccharide ions had a higher mobility than those of dextran as expected and the difference increased as the DP increased. For example, there was essentially no difference in the mobility of the singly charged compounds with DP 2 but a significant difference was observed with DP >6 when helices would be expected to form in the maltooligosaccharides (Table 2). The one exception to the order of mobility was with the compound with DP 3 where the mobility of the dextran was higher than that of the maltooligosaccharide. With the doubly charged species, which have more extended conformations due to charge repulsion, the difference was most discernible with DP ≥11 (Table 3) implying the distortion of different inherent conformations became less significant as intra-molecular charge repulsion weakened in longer oligomers. The separation of ions with DP ≥16 and up to 35 was readily observed in the triply charged state (Table 4 and Fig. 2). The sensitivity of this approach is exemplified by the limited solubility of maltooligosaccharides with a DP of >18.^32^ Thus, it was indeed possible to discriminate between the conformations of maltooligosaccharides and dextrans with a wide range of DP and high sensitivity using IMMS.

**Table 2:** Negative ion mobility and collision cross-section (Ω) of singly charged ions of maltooligosaccharides, dextran and pullulan

		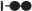		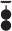		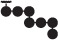	
DP	*m/z*	maltooligosaccharide		dextran		pullulan	
		drift time (ms)	Ω (Å^2^)	drift time (ms)	Ω (Å^2^)	drift time (ms)	Ω (Å^2^)
2	341.1	2.95	115	3.09	118	3.02	116
3	503.2	4.46	144	4.32	141	4.32	141
4	665.2	5.22	159	5.64	167	5.64	167
5	827.3	6.74	188	7.01	194	7.01	194
6	989.3	7.80/8.19	209/217	8.54	223	8.54	223
7	1151.4	9.51	242	9.85	249	9.85	249
8	1313.5	10.60	263	11.37	278	11.30	277
9	1475.5	11.57	282	12.75	305	12.68	304
10	1637.5	12.61	302	13.88	327	13.58	321

Maltooligosaccharides in amylose 2800 are α-1,4-linked glucans with a range of DP; both isomers detected with DP 6 are shown; note the drift times of ions within the mixture are 1–2% lower than those with pure compounds (Table 1).

Dextran 5000 is an α-1,6-linked glucan with a range of DP.

Pullulan comprises repeating units of α-1,4-linked maltotriose connected by α-1,6 linkages; the pullulan 1300 sample contained a range of DP.

Singly charged α,ω-dicarboxy-terminated polystyrene was used as a calibrant.

**Table 3:** Negative ion mobility and collision cross-section (Ω) of doubly charged ions of maltooligosaccharides, dextran and pullulan

		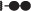		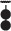		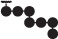	
DP	*m/z*	maltooligosaccharide		dextran		pullulan	
		drift time (ms)	Ω (Å^2^)	drift time (ms)	Ω (Å^2^)	drift time (ms)	Ω (Å^2^)
3	251.1			1.91	161		
4	332.1			2.32	169	2.19	177
5	413.1			2.74	209	2.74	209
6	494.2	3.01	225	2.94	221	2.94	221
7	575.2	3.21	237	3.35	245	3.35	245
8	656.2	3.49	253	3.77	270	3.77	270
9	737.3	3.91	278	4.18	293	4.18	293
10	818.3	4.31	301	4.52	313	4.52	313
11	899.3	4.31	301	4.87	334	4.87	334
12	980.3	4.73	325	5.28	358	5.28	358
13	1061.4	5.07	345	5.63	378	5.63	378
14	1142.4	5.35	362	5.97	398	5.97	398
15	1223.4	5.69	381	6.38	422	6.31	418
16	1304.4	6.10	405	6.66	438	6.52	430
17	1385.5	6.52	444	6.80	446	6.73	442
18	1466.5	6.87	450	7.28	474	7.14	466
19	1547.5	7.21	470	7.56	490	7.56	490
20	1628.6	7.56	490	8.04	518	7.97	514
21	1709.6	7.82	506	8.45	542	8.38	538
22	1790.6	8.24	530	8.86	566	8.86	566
23	1871.7	8.66	555	9.35	595	9.28	591
24	1952.7	8.79	562	9.76	619	9.69	615

Maltooligosaccharides in amylose 2800 are α-1,4-linked glucans with a range of DP.

Dextran 5000 is an α-1,6-linked glucan with a range of DP.

Pullulan comprises repeating units of α-1,4-linked maltotriose connected by α-1,6 linkages; the pullulan 1300 sample contained a range of DP.

Doubly charged α,ω-dicarboxy-terminated polystyrene was used as a calibrant.

**Table 4:** Negative ion mobility and collision cross-section (Ω) of triply charged ions of maltooligosaccharides and dextran

		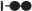		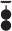	
DP	*m/z*	maltooligosaccharide		dextran	
		drift time (ms)	Ω (Å^2^)	drift time (ms)	Ω (Å^2^)
14	761.6			3.49	376
15	815.3			3.69	390
16	869.6	3.34	362	3.76	395
17	923.6	3.48	373	3.97	410
18	977.6	3.69	380	4.11	419
19	1031.6	3.90	404	4.31	433
20	1085.6	4.04	413	4.52	447
21	1139.6	4.11	418	4.66	456
22	1193.4	4.31	431	4.87	470
23	1247.4	4.44	440	5.06	483
24	1301.4	4.58	450	5.20	492
25	1356.1	4.72	459	5.48	510
26	1410.2	4.93	473	5.62	520
27	1463.8	5.13	486	5.83	532
28	1523.5	5.48	509	5.96	540
29	1577.5	5.69	523	6.17	554
30	1631.5	5.90	536	6.38	567
31	1685.5	6.03	544	6.59	580
32	1740.2	6.23	557	6.72	587
33	1794.0	6.44	570	6.92	599
34	1848.1	6.65	583	7.20	616
35	1902.0	6.92	599	7.41	629

Maltooligosaccharides in amylose 2800 are α-1,4-linked glucans with a range of DP.

Dextran 5000 is an α-1,6-linked glucan with a range of DP.

Singly and doubly charged α,ω-dicarboxy-terminated polystyrene was used as a calibrant with the Driftscope software extrapolating the triply charged calibration curve.

### IMMS separation of linear homo- and hetero-oligomeric glucans

Pullulan is a hetero-oligomeric glucan and comprises repeating units of α-1,4-linked maltotriose connected by α-1,6 linkages.^4^ Its biosynthesis involves the formation of the α-1,6 linkage first, so its component compounds with DP >2 contain both glycosidic linkages. Pullulan has a random coil structure like that of dextran due to the presence of the α-1,6 linkages.^33^ Thus it would be expected that hetero-oligomeric pullulan would have similar ion mobility to that of homo-oligomeric dextran. The IMMS of pullulan oligosaccharides showed that this was indeed the case in their singly and doubly charged states (Tables 2 and 3).

The component of pullulan with a DP of 2 would be expected to have an α-1,6 linkage and thus be identical to the corresponding component of dextran (i.e. isomaltose). The measured difference between the drift times of these components was ∼2% and within the error observed when complex mixtures of oligosaccharides were analysed. However, it is possible that the pullulan sample contained a sufficient amount of the α-1,4-linked isomer, maltose, to distort the drift time without allowing the two components to be resolved.

**Figure 3:**
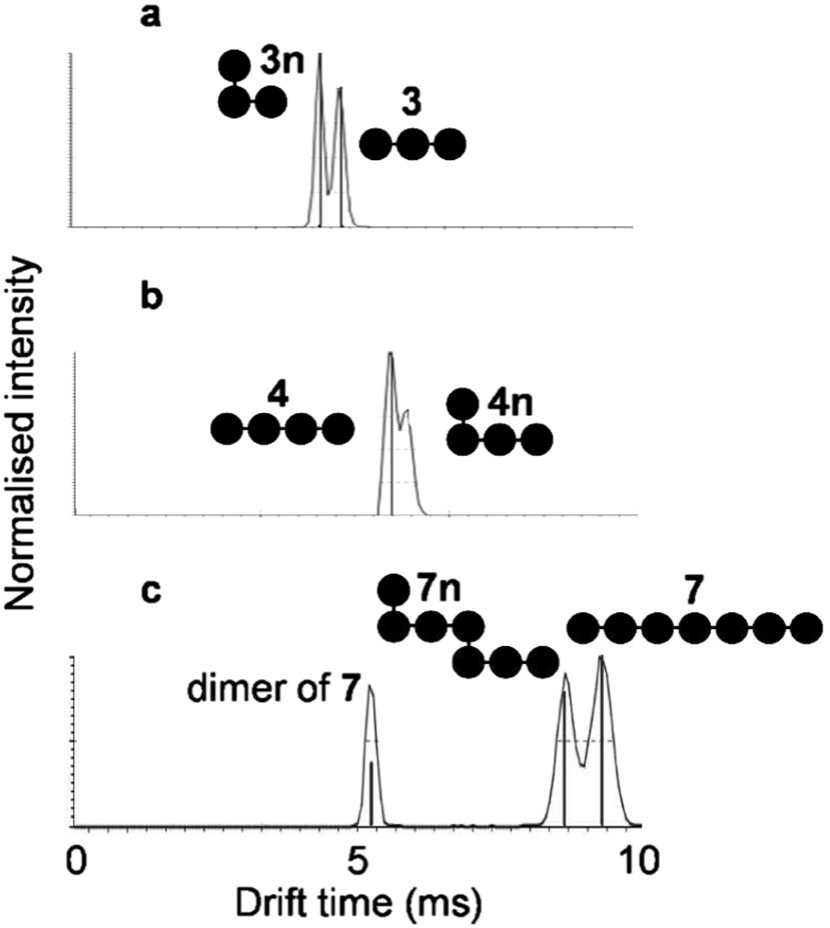
IMMS of isomeric α-glucans with DP 3 (a, *m/z* 503.2; 3 and 3n), DP 4 (b, *m/z* 665.2; 4 and 4n) and DP 7 (c, *m/z* 1151.4; 7, including its doubly charged dimer, and 7n).

The IMMS of specific authentic hetero-oligomeric oligosaccharides **3n**, **4n** and **7n** was then compared with the corresponding homo-oligomeric maltooligosaccharide isomers **3**, **4** and **7** (Table 1 and Fig. 3). Given the observations made with pullulan, it was expected that **3n** and **4n** would have had higher and lower mobilities compared with **3** and **4**, respectively. This was indeed the case. Unexpectedly, **7n** had a lower ion mobility than **7**. Co-injection of **7n** with pullulan allowed the discrimination of two species with DP 7 by IMMS (data not shown), confirming that the structure of the DP 7 compound in pullulan was not that of **7n**. The difference is likely due to the presence of an α-1,6 linkage at the reducing end of the DP 7 compound in pullulan. Therefore, it is possible to distinguish between maltooligosaccharides and either dextran or hetero-oligomeric pullulan. Furthermore, it is also possible to distinguish, at least in one case, between hetero-oligomeric isomers such as **7n** and pullulan with DP 7, consistent with the biosynthesis of pullulan occurring through a specific sequence of elongation steps.^4^

### Isomers separated by mobility fragment differently

**Figure 4:**
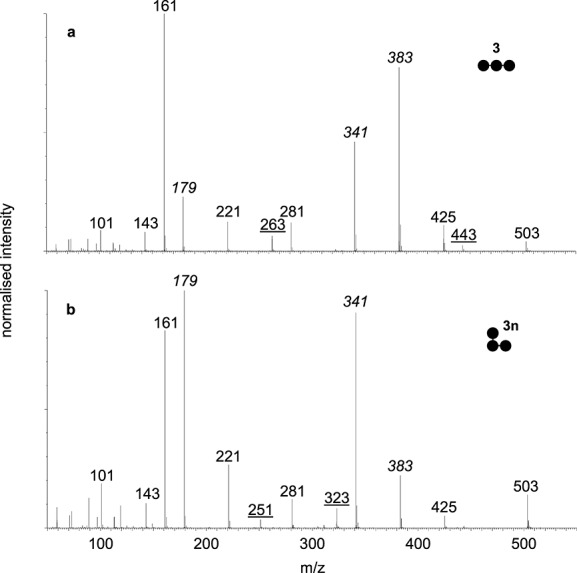
Fragmentation of 3 (a) and 3n (b) oligosaccharides with DP 3 after separation by IMMS. Ions that are specific to an isomer are underlined and ions that have significantly different abundances are in *italics*. Proposed assignments of ions are shown in Supplementary Fig. S1 (see Supporting Information).

The combination of IMMS and tandem mass spectrometry has been demonstrated to add additional analytical information,^14^,^17^,^19^ so this approach was used with some homo- and hetero-oligomeric glucans. Compounds **3** and **3n** fragmented differently (Fig. 4) and it was possible to assign all major fragment ions (Supplementary Fig. S1, see Supporting Information). Some of the less abundant ions were more strongly associated with one compound. For example, **3** gave a 263 *m/z* ion and loss of 60 to 443 *m/z* while **3n** gave 251 and 323 *m/z* ions. The most significant differences were in the relative abundances of the 179, 341 and 383 *m/z* ions. Different fragmentation spectra were also obtained with the corresponding compounds with DP 4 and 7 (Supplementary Figs. S2, S3, S4 and S5, see Supporting Information). Interestingly, similar unique ions were observed with the compound with DP 4. For example, **4** gave a 263 *m/z* ion and loss of 60 to 605 *m/z* while **4n** gave 251 and 323 *m/z* ions. These unique ions presumably reflect the different glycosidic linkage at the non-reducing ends of these compounds. However, these ions were not observed in all cases because they were not detected with the compounds with DP 7. The α-1,6-glycosidic linkage is known to be stronger than the α-1,4 linkage,^34^ and this might explain the fewer number of fragment ions derived from the larger hetero-oligomeric compounds **4n** and **7n** compared with their counterparts **4** and **7**. Such diagnostic fragmentation patterns do indeed provide the opportunity to further distinguish between isomers, ideally when authentic standards are available.

### IMMS can be used to detect GlgB branching enzyme activity

**Figure 5:**
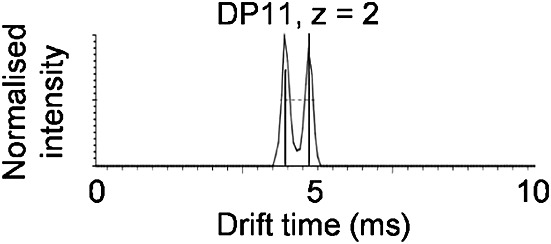
IMMS of two isomers of α-glucan with DP 11 (*m/z* 899, z = 2) within amylose 2800 treated with *M. tuberculosis* GlgB branching enzyme. The ion with the lower drift time was present before treatment with GlgB and the ion with the higher drift time appeared after treatment.

In order to assess whether IMMS of oligosaccharides could be used to analyse enzyme-catalysed reactions in complex biological samples, amylose 2800 was treated with the genetically validated anti-tuberculosis drug target *M. tuberculosis* GlgB (EC 2.4.1.18).^35^ GlgB is a transglucosylating branching enzyme that transfers a portion of linear maltooligosaccharide onto the 6 position of a glucose ring within a maltooligosaccharide chain to give a branched product.^36^ Given the substrates and products of GlgB activity are isobaric, IMMS could allow their discrimination. Indeed, the formation of a new doubly charged species with DP 11 of lower mobility than the corresponding linear substrate isomer was detected after treatment with GlgB (Table 1 and Fig. 5). The lower mobility of the branched product is consistent with the higher hydrodynamic radius of branched isomers^25^ and other IMMS studies with branched oligosaccharides.^13^ The inability to detect any new isomers with a lower DP suggests that the smallest branched product that this enzyme can generate has a DP of 11.

## CONCLUSIONS

It is possible to resolve isobaric linear glucans with a DP of up to 35 using ESI travelling-wave IMMS in the negative mode. The degree of separation depends on the type of α-glycosidic linkages, the DP and the charge state of the ions. Importantly, the increased conformational freedom of α-1,6 over α-1,4 linkages observed in solution was retained in the gas phase, allowing large linear oligosaccharides to be distinguished. The differential fragmentation of linear homo- and hetero-oligomeric α-glucans separated by IMMS added another level of diagnostic potential with this approach. The CCSs of α-glucan ions in the negative mode were determined for the first time using α,ω-dicarboxy-terminated polystyrene as a calibrant. The accumulation of more data of this type could underpin a database allowing structural information to be gleaned from using this approach. Two conformational isomers of maltohexaose were separable by IMMS, presumably due to its ability to form both extended and V6 helical conformations. Importantly, the ability to detect products of a potential anti-tuberculosis drug target, GlgB branching enzyme, in a complex mixture has been demonstrated using IMMS. This work paves the way for other biological samples of complex isobaric oligosaccharides to be analysed using IMMS in the negative mode providing quick analyses and high sensitivity without the need for either derivatisation or chromatographic separation. This could help elucidate the composition, biosynthesis, modification and degradation of α-glucans and have utility with other α-glucans with α-1,2 and α-1,3 linkages and other large oligosaccharides.
