# Supplementary material for: Discrimination of large maltooligosaccharides from isobaric dextran and pullulan using ion mobility mass spectrometry
Source: Rapid Commun Mass Spectrom. 2014 Dec 5;28(2):191–9. doi: 10.1002/rcm.6771 (PMC4285287; doi:10.1002/rcm.6771)
Supplement: Supplementary file 1 [file rcm0028-0191-sd1.docx]

**Supporting Information**

**Discrimination of large maltooligosaccharides from isobaric dextran and pullulan using ion mobility mass spectrometry**

Short title: Ion mobility mass spectrometry of large isobaric alpha-oligoglucans

**Abdul M. Rashid, Gerhard Saalbach and Stephen Bornemann***

Department of Biological Chemistry, John Innes Centre, Norwich Research Park, Norwich NR4 7UH, United Kingdom

* *Correspondence to:* S. Bornemann

E-mail: [stephen.bornemann@jic.ac.uk](mailto:stephen.bornemann@jic.ac.uk)

Tel: +44 (0)1603 450741

Fax: +44 (0)1603 450018

**Table S1.** Negative ion mobility of α,ω-dicarboxy-terminated polystyrene for the calibration of collisional cross section (Ω)

| z | m/z | drift time (ms) | Ω (Å^2^)  Literature value |
| --- | --- | --- | --- |
| 1 | 401.20 | 4.19 | 132 |
| 1 | 505.25 | 5.36 | 163 |
| 1 | 609.33 | 6.68 | 196 |
| 1 | 713.40 | 7.99 | 210 |
| 1 | 817.47 | 9.09 | 236 |
| 1 | 921.54 | 10.34 | 262 |
| 1 | 1025.60 | 11.58 | 276 |
| 2 | 304.17 | 2.60 | 211 |
| 2 | 356.20 | 3.02 | 235 |
| 2 | 408.24 | 3.43 | 239 |
| 2 | 460.27 | 3.92 | 265 |
| 2 | 512.30 | 4.25 | 291 |
| 2 | 564.34 | 4.67 | 317 |
| 2 | 616.37 | 5.02 | 335 |
| 2 | 668.40 | 5.36 | 375 |
| 2 | 720.44 | 5.71 | 388 |

J.V. Hamilton, J.B. Renaud, P.M. Mayer. Experiment and theory combine to produce a practical negative ion calibration set for collision cross-section determinations by travelling-wave ion-mobility mass spectrometry. *Rapid Commun. Mass Spectrom.* **2012**, 26, 1591.

**Table S2.** Negative ion mobility and collision cross section (Ω) of oxidised insulin chain A from bovine pancreas (most abundant theoretical mass, 2,530.9 Da)

| z | m/z | drift time (ms) | Ω (Å^2^) | |
| --- | --- | --- | --- | --- |
|  |  |  | This work | Literature value |
| 2 | 1264.5 | 6.80 | 387 | 388 |
| 3 | 842.6 | 3.62 | 391 | 390 |
| 4 | 631.7 | 3.28 | 474 | 470 |
| 5 | 505.0 | 2.94 | 562 | Not reported |

Note that the Driftscope software extrapolated CCS calibration curves for ions with z ≥3 from the calibration curves obtained with z = 1 and z = 2.

A.E. Counterman, S.J. Valentine, C.A. Srebalus, S.C. Henderson, C.S. Hoaglund, D.E. Clemmer. High-order structure and dissociation of gaseous peptide aggregates that are hidden in mass spectra. *J. Am. Soc. Mass Spectrom.* **1998**, 9, 743.

**Table S3.** Negative ion mobility and collision cross section (Ω) of decadeoxythymidine (monoisotopic theoretical mass, 2978.5 Da)

| z | m/z | drift time (ms) | Ω (Å^2^) | |
| --- | --- | --- | --- | --- |
|  |  |  | This work | Literature value |
| 2 | 1488.3 | 7.77 | 421 | 447 |
| 3 | 991.9 | 4.38 | 440 | 446 |
| 4 | 743.7 | 4.05 | 537 | 537 |
| 5 | 594.7 | 3.42 | 618 | 627 |
| 6 | 495.4 | 2.73 | 650 | 641 |

The triply charged ion was the most abundant. Note that the Driftscope software extrapolated CCS calibration curves for ions with z ≥3 from the calibration curves obtained with z = 1 and z = 2.

C.S. Hoaglund, Y.S. Liu, A.D. Ellington, M. Pagel, D.E. Clemmer. Gas-phase DNA: oligothymidine ion conformers. *J. Am. Chem. Soc.* **1997**, 119, 9051.

**Figure S1.** Proposed assignment of fragment ions (each with loss of 1H^+^) associated with compounds **3** and **3n** (parent ion m/z 503) as shown in Figure 4. Not all potential means of arriving at each ion are shown. Ions that are specific to an isomer are underlined and ions that have significantly different abundances are in *italics*. The nomenclature of Domon and Costello has been used.

B. Domon, C.E. Costello. A systematic nomenclature for carbohydrate fragmentations in FAB-MS/MS spectra of glycoconjugates. *Glycoconjugate J.* **1988**, 5, 397.

**Figure S2.** Fragmentation of **4** (a) and **4n** (b) oligosaccharides with DP4 after separation by IMMS. Ions that are specific to an isomer are underlined.

**Figure S3.** Proposed assignment of fragment ions (each with loss of 1H^+^) associated with compounds **4** and **4n** (parent ion m/z 665) as shown in Figure S2. Ions that are specific to an isomer are underlined. Not all potential means of arriving at each ion are shown.

**Figure S4.** Fragmentation of **7** (a) and **7n** (b) oligosaccharides with DP7 after separation by IMMS.

**Figure S5.** Proposed assignment of fragment ions (each with loss of 1H^+^) associated with compounds **7** and **7n** (parent ion m/z 1151) as shown in Figure S4. Not all potential means of arriving at each ion are shown.
